# Supplementary figures and images for: Porcine Deltacoronavirus Utilizes Sialic Acid as an Attachment Receptor and Trypsin Can Influence the Binding Activity
Source: Viruses. 2021 Dec 6;13(12):2442. doi: 10.3390/v13122442 (PMC8705999; doi:10.3390/v13122442)

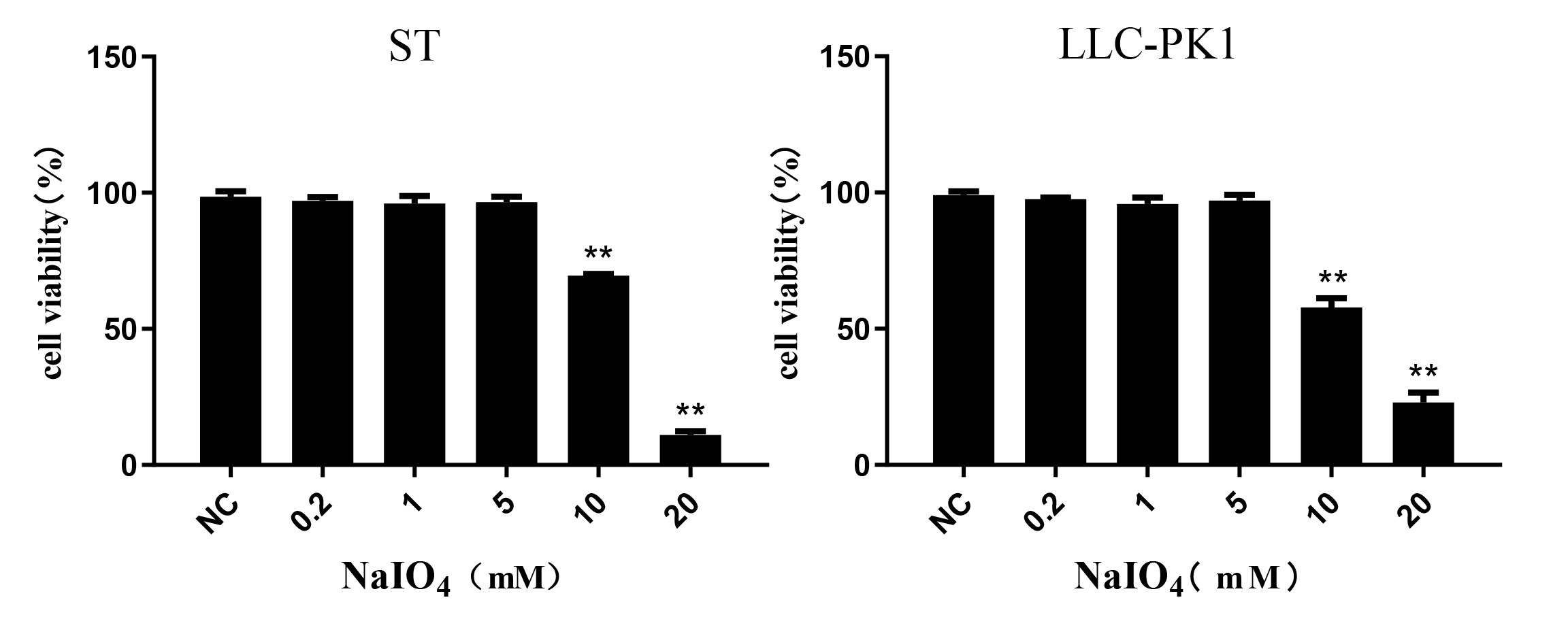

Supplement: Supplementary file 1 [file viruses-13-02442-s001.zip › Fig.S1.tif]

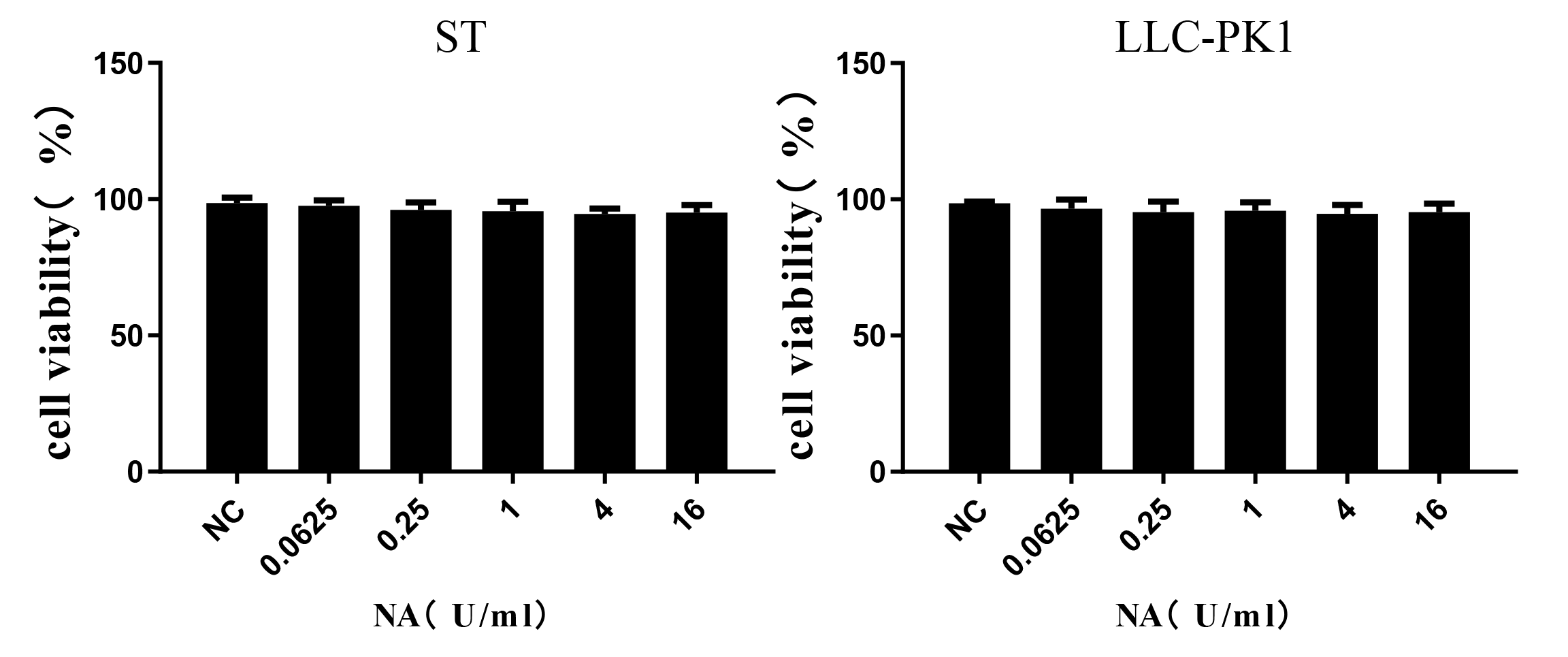

Supplement: Supplementary file 1 [file viruses-13-02442-s001.zip › Fig.S2.tif]
